# Aging well in an aging society: physical health in older lesbian, gay, and bisexual adults

**DOI:** 10.3389/fpsyg.2024.1369021

**Published:** 2024-05-27

**Authors:** Roberto Baiocco, Chiara Antoniucci, Jessica Pistella, Guido Alessandri, Fabio Alivernini, Anna M. Borghi, Andrea Chirico, Lorenzo Filosa, Chiara Fini, Tommaso Palombi, Fau Rosati, Simone Tavolucci, Fabio Lucidi

**Affiliations:** ^1^Department of Developmental and Social Psychology, Faculty of Medicine and Psychology, Sapienza University of Rome, Rome, Italy; ^2^Department of Psychology, Sapienza University of Rome, Rome, Italy; ^3^Department of Dynamic, Clinical Psychology and Health, Faculty of Medicine and Psychology, Sapienza University of Rome, Rome, Italy

**Keywords:** older LGB, physical health, aging well, discrimination, coming out, resiliency

## Abstract

**Introduction:**

Older sexual minority people meet a double stigma in our society related to their sexual identity and chronological age. The present study explores how experiences of discrimination and prejudice, coming out, and personal resiliency influence physical health of older lesbian, gay, and bisexual (LGB) adults.

**Methods:**

Respondents were recruited through online advertisements and an online-based survey. The sample included 82 Italian cisgender LGB adults over 65 years: young older adults (65–70 years; 78%) and old-old adults (over 71 years; 22%). Regarding sexual orientation, the sample was composed of sexual minority women (*n* = 30; 37%) and sexual minority men (*n* = 52; 63%).

**Results:**

ANOVAs’ findings showed that sexual minority women described lower levels of physical health compared to sexual minority men. At the same time, old-old adults reported higher experiences of discrimination and prejudice compared to young older adults. Moreover, findings from hierarchical multiple regression analysis described that coming out, higher levels of personal resiliency, and fewer experiences of discrimination were predictors of physical health, regardless of age and sexual minority categories.

**Conclusion:**

These findings seem to align with previous studies that underline the relevance of investigating aging well in sexual minority people. Knowledge and awareness of LGBTQ+ issues are necessary for recognizing the unique needs and resources of older LGB people for promoting a healthy aging process.

## Introduction

The experience of marginalization and invisibility of older lesbian, gay, bisexual, and other non-heterosexual people have been explored in the literature mainly in the last decades (D’Augelli et al., 2001; D’Augelli and Grossman, 2001; Fredriksen-Goldsen and Muraco, 2010; Fredriksen-Goldsen et al., 2013; Caceres et al., 2017; Fredricksen-Goldsen and de Vries, 2019; Kendrick et al., 2021). Notably, a particular line of research investigated the experiences of victimization and vulnerability in specific social settings, like physical activity-related contexts, focusing on the relationships between social invisibility, minority stressors, resilience dimensions, and physical health of older and young sexual and gender minority adults (Fredriksen-Goldsen et al., 2015, 2017; Ávila et al., 2018, 2021; Baiocco et al., 2018a,b; Kendrick et al., 2021; Pistella et al., 2024). Thus, the present study will explore how experiences of coming out, minority stress, and personal resiliency could have an impact on the physical health of older lesbian, gay, and bisexual (LGB) adults.

Older sexual minority people meet a double stigma in our society related to their sexual identity (Baiocco et al., 2022) and chronological age (Iversen et al., 2009). Indeed, within the aging population, older sexual minority adults face unique challenges and discrepancies due to their sexual orientation. Different studies have underscored the heightened risks this demographic encounters in terms of both physical and mental well-being, primarily attributed to the historical context in which they lived, marked by societal discrimination and the criminalization of homosexuality (D’Augelli et al., 2001; Scandurra et al., 2017; Almack and King, 2019; Rosati et al., 2021a; Pistella et al., 2024).

Notably, the sum of these forms of discrimination was articulated by Meyer (2003) within the minority stress model, which posits that health inequities among sexual minorities stem from increased exposure to social stressors, given their marginalized status compared to heterosexual individuals. The minority stress model identifies two categories of stressors: (1) distal stressors arising from institutional and interpersonal discrimination and (2) proximal stressors related to internalized stigma, anticipation of rejection, and concealment of sexual orientation. Despite being formulated over two decades ago, Meyer’s model remains pertinent, with recent adaptations focusing on physiological stress pathways and specific minority stressors that sexual and gender minority people encounter within cis-hetero-normative societies (Frost and Meyer, 2023). Accordingly, with Meyer’s model (2003), some studies highlighted that older sexual minority people reported a higher level of internalized sexual stigma compared to their younger counterparts, probably due to past episodes of discrimination and the lack of rights and recognition that characterized past society (Denison and Kitchen, 2015; Fredricksen-Goldsen and de Vries, 2019; Rosati et al., 2020). Moreover, several studies highlighted those experiences of discrimination and stigma (i.e., internalized sexual stigma) could predict lower levels of physical health and general health disparities between older LGB population and older heterosexual people (Dilley et al., 2010; Scandurra et al., 2017; Fredricksen-Goldsen and de Vries, 2019; Kendrick et al., 2021; Pistella et al., 2024).

The interpretation of “health” regarding old age is open to discussion. It is widely agreed upon that merely lacking diseases does not fully capture health in old age due to the high occurrence of identifiable health issues among older people (Lawton and Lawrence, 1994; Smith, 2001; Smith et al., 2002; Schöllgen et al., 2016). Instead, according to the literature, at this age, health is seen as a multi-dimensional construct: alongside diagnosing diseases, it involves evaluating discomfort linked to symptoms (like pain), potential risks to life, repercussions of treatments (such as medication side effects), functional abilities, and personal evaluations of health (Lawton and Lawrence, 1994; Borchelt et al., 1999). Moreover, considering the general picture that older adults of today are healthier than in the past because there have been improvements in most dimensions of health (i.e., mortality, risk factors, disease prevalence, and incidence), several studies investigated perspectives of aging well and new trajectories of development through this specific age (Crimmins, 2004).

According to Rowe and Kahn (1997), successful aging is more than the absence of diseases; it refers to three main different elements: (1) moderate likelihood of disease and disease-related disability, referring not only to the absence of illness itself but also to the risks factors to disease; (2) high cognitive and physical abilities, which are related not to what a person does do, but to what an older adult can do; (3) active engagement with life, which refers to many forms of engagement, as interpersonal relation and productive activity. Indeed, one of the elements that literature recognized as necessary in aging well is doing sport and physical activity in older age (e.g., Slevin, 2010). The World Health Organization (WHO) prescribed a min of 150 min of moderate PA and/or 75 min of vigorous PA a week for older adults to reduce the risks of physical disease (e.g., cardiovascular heart disease; Hasson et al., 2017).

Several studies demonstrated that physical activities (PA) and sporting behaviors are also crucial for improving happiness and resilience to mental health disorders in older age, promoting psychological well-being and general aging well (Richards et al., 2015; Ávila et al., 2018, 2021; Martínez-Moreno et al., 2020). Notably, a recent study by Martínez-Moreno et al. (2020) showed that older adults who participated in PA (e.g., swimming, soft trekking, walking, or yoga) reported higher levels of optimism, resilience, and engagement in groups of other older adults. Similar results were found in a study by Pharr et al. (2021) on sexual minority adults, highlighting that participants who practiced PA reported higher mental health in terms of fewer daily experiences of depression, anxiety and/or problems with emotions compared to the other that did not practice any PA.

Moreover, a specific line of research investigated the engagement of sexual minority young and older adults in sports or PA, showing opposite results: some studies reported that, on average, sexual minority people spent less time in regular exercise compared to heterosexual individuals, consequently falling into risks of poor general health, obesity, disabilities, and depression (Abichahine and Veenstra, 2017; Cunningham et al., 2018; Lindström and Rosvall, 2020). On the other side, different studies highlighted no significant differences in PA between sexual minority people and heterosexual individuals both in older age and younger age (Dilley et al., 2010; Nelson, 2023).

Simultaneously, other studies highlighted that the experiences of discrimination, rejection, and prejudice (e.g., *distal minority stressors*) could play a role in less engagement in regular exercise among sexual minority women (Mereish and Goldstein, 2020; Pharr et al., 2021). Whereas stigma, fear of rejection, or concealed one’s sexual orientation (e.g., *proximal stressors*) were indirectly related to lower odds of reporting regular exercise and indirectly determined more excess body fat (Mereish and Goldstein, 2020; Pharr et al., 2021).

Additionally, studies that investigated health and well-being in older sexual minority adults reported differences related to gender and sexual orientation: generally, gay men had reported higher levels of engagement in PA compared to lesbian women and bisexual individuals according to the desire to be more physically attractive (Slevin and Linneman, 2010; Zhang et al., 2017; Lightner et al., 2020). Indeed, a set of 10 interviews conducted on old gay men by Slevin and Linneman (2010) pointed out that old gay men spent more time caring for their bodies, doing sports or PA to improve their physical health, and keeping their bodies youthful through exercise, compared to others sexual minority people and heterosexual individuals. Again, differences were found in the sexual minority old age groups: Fredriksen-Goldsen et al. (2017) found that the percentage of PA in young-old and middle-old adults was higher compared to the old age group, which reported being more involved in leisure activities.

### The present study

The present study aims to explore how coming out, personal resiliency and experiences of discrimination and prejudice could predict physical health in a group of older Italian LGB adults. Moreover, even though in recent years, there has been a proliferation of studies regarding the health and well-being of older sexual minority people, the majority of these were conducted in the U.S. (Fredricksen-Goldsen and de Vries, 2019). At the same time, just a few belong to other Countries, like Italy, where getting older the population and the resistance to prejudice and discrimination against the LGB community are relevant issues (Baiocco et al., 2022; Pistella et al., 2023). Notably, the Italian context is still resistant to the recognition and affirmation of LGB people in general, and specifically older sexual minority adults (Rosati et al., 2021a). Thus, considering the higher level of invisibilization and the necessity of hiding one’s own sexual identity within social contexts, encountering this population in the Italian context could be quite challenging.

Accordingly, starting from the literature that highlighted how the experience of invisibility distal and proximal minority stressors impacted older sexual minority adults’ physical health, we also considered the relevance of personal resiliency to reduce stressors and promote well-being (Richards et al., 2015; Ávila et al., 2018, 2021; Martínez-Moreno et al., 2020). Additionally, through physical health, we explored not only participants’ physical disease or the impact of their disabilities on their everyday lives but also the degree of engagement in PA. Indeed, it could represent a protective factor for aging, promoting physical health in older LGB people (Slevin, 2010; Pharr et al., 2021).

## Method

### Procedures

Participant recruitment and data collection were conducted between April 2023 and November 2023 as part of the national research project “Ageing Well in an Ageing Society,” funded by Next Generation EU [DM 1557 11.10.2022], in the context of the PNRR, Investment PE8—Project Age-It. A specific research line of the project aimed to highlight how the coming out process, personal resiliency, and experiences of direct/indirect discrimination could predict physical health in a group of 82 older LGB adults.

Respondents were recruited through online advertisements and an online-based survey (hosted by Unipark). Notably, participants were reached through different ways: (1) personal networks of the research groups; (2) Italian associations of older sexual minority adults (Agapanto Association; https://anzianilgbt.blogspot.com); (3) advertisements on leading social networks (e.g., Facebook and Instagram). Participation in the study was voluntary and anonymous, and before the questionnaire, each participant received informed consent to participate. All respondents answered the same questionnaires that required approximately 25–30 min to complete the online survey (95% completed the questionnaire).

The online survey aligned with the Checklist for Reporting Results of Internet E-Surveys (CHERRIES; Eysenbach, 2004). Before initiating the data collection, the research protocol was approved by the project coordinator’s Ethics Committee and most project partners in their local organization. All procedures performed with human participants were conducted following the ethical standards of the institutional and national research committee and with the 2013 Declaration of Helsinki or comparable ethical standards. Data are available upon request in compliance with the General Data Protection Regulation (GDPR; Regulation, 2016) on the protection of natural persons regarding the processing of personal data and the free movement of such data.

### Participants

A power analysis (alpha = 0.05 and power = 0.80) showed that the projected minimum sample size needed to detect an effect size of *f* = 0.15 is *n* = 77 (for regression analysis, using G*Power 3.1; Faul et al., 2007). The sample included 82 cisgender LGB adults over 65 years. Regarding sexual orientation, considering the numerosity of the sample, we decided to split it into two groups: (a) sexual minority women (*n* = 30; 37%), which comprised both lesbian women (*n* = 22; 73%), and bisexual women (*n* = 8; 27%), and (b) sexual minority men (*n* = 52; 63%), which included both gay men (*n* = 40; 77%), and bisexual men (*n* = 12; 23%). Moreover, according to the literature (Fredriksen-Goldsen et al., 2015, 2017), we divided the sample into two age groups: Young older adults (65–70 years; *n* = 64, 78%) and old-old adults (over 71 years; *n* = 18; 22%). Finally, regarding Social Economic Status (SES), 1,2% of participants were in an extremely low condition, 15,9% were in a low condition, 70,7% reported having an average condition, 11% had a high condition, and 1,1% reported a very high SES.

### Measures

#### Sociodemographic variables

Baseline sociodemographic variables such as age, SES, and biological sex (0 = female, 1 = male) were asked at first.

Sexual identity was asked through two different questions that investigated older adults sex assigned at birth (1 = female; 2 = male; 3 = intersex) and respondents’ gender identity (0 = cisgender woman; 1 = cisgender man; 2 = transgender woman; 3 = transgender man; 4 = non binary person; 5 = other; 6 = I prefer to not specify).

#### Coming out

Coming out (CO) was asked through a question (Rosati et al., 2020) that investigated the percentage of people who know participants’ sexual orientation, rating from 1 (“0%”) to 5 (“100%”).

#### Physical health

We used the scale of physical health from the study of D’Augelli et al. (2001) that aims to assess the physical health among older lesbian, gay, and bisexual adults. Physical health was explored through five questions that investigated: (1) the current overall physical health (“*How would you describe your physical health at present?*”); (2) how health interfered with daily activities (“*How often does your physical health stand in the way of doing the things you want to do?*”); (3) the changes of physical health in the past 5 years (“*Generally, how is changed your physical health in the past 5 years?*”); (4) the change in physical activities in the past 5 years (“*How has your ability to perform physical activities such as walking, shopping, and working around the house, changed in the past five years?*”); (5) finally, the frequency of participation in physical activities (“*How frequently do you do physical activities as jogging, excursions, swimming, mountain bike or walking*?”). At each question participants answered through a 5-point scale from 1 (“Much worse”) to 5 (“Much better”). The alpha reliability was 0,74.

#### The daily heterosexist experiences questionnaire

Experiences of prejudice and discrimination were measured with a reduced version of four subscales of the questionnaire: (1) Isolation (4 items; “*Difficulty finding a partner because you are LGBT*”); (2) Vigilance, which captures proximal stressors that reflect will to hide one’s sexual orientation (6 items; “*Watching what you say and do around heterosexual people*”); (3) Gender Expression, which refers to proximal stressors too, and considered both experiences of mistreatment, discrimination and discomfort related to one’s gender expression (6 items; “*Being misunderstood by people because of your gender expression*”); (4) Discrimination/Harassment, which refers to distal stressors, and refers to experiences of discrimination and teasing related to one’s sexual orientation (5 items; “*Being verbally harassed by strangers because you are LGBT*”); (5) Victimization, which captures distal stressor as well, related to experiences of direct physical and sexual aggressions (“*Being punched, hit, kicked, or beaten because you are LGBT*”). Experiences of prejudice and discrimination were measured with a reduced version (Balsam et al., 2013). For each dimension, participants responded to “*How much has this problem distressed or bothered you during the past 12 months*?” using the following response categories 1 (“Did not happen/not applicable to me”), 2 (“It happened and it bothered me not at all”), 3 (“It happened, and it bothered me a little bit”), 4 (“It happened, and it bothered me moderately”), 5 (“It happened, and it bothered me quite a bit”), 5 (“It happened, and it bothered me extremely”). The alpha reliability was 0,79.

#### Resilience scale—personal competence

Resilience scale—personal competence (RSCS; Wagnild and Young, 1993) is a 25-item self-report scale that measures the degree of individual resilience considered a positive personality characteristic that could moderate the effect of stress. Moreover, this scale was developed within a sample of older adults. Notably, the present study considered just the items of the Personal Competence’s dimension, reflecting self-reliance, independence, determination, invincibility, mastery, resourcefulness, and perseverance (“*I am determined*”). For each question, participants could answer from 1 (“Completely Disagree”) to 7 (“Completely Agree”). The alpha reliability was 0,72.

### Data analysis

Statistical Package for the Social Sciences (SPSS 25.0) was used for bivariate and multivariate analyses. Preliminary descriptive statistics, ANOVAs, and bivariate correlations were examined to explore gender differences (i.e., sexual minority women VS sexual minority men), age groups (i.e., young-old adults VS old-old adults), and the associations among the key variables. In addition, we investigated the percentage of the coming out, taking into account gender and sexual orientation: We performed contingency table tests, a chi-square test of association, and considered the adjusted standardized residuals, referring to the percentage of coming out within the sexual minority men and sexual minority women’s groups. Internal consistency was measured using Cronbach’s *α*. Moreover, hierarchical multiple regression was conducted to test the effects of coming out, experiences of discrimination and prejudice (i.e., DHEQ questionnaire), and personal resiliency (i.e., RSCS) on physical health. Before regression analyses were performed, linearity, homoscedasticity, normality of residuals, and multicollinearity assumptions were assessed. Dependent and continuous variables were standardized before analysis.

## Results

### Differences between groups among age and gender

One-way ANOVAs were conducted to test for mean differences in the study variables (Tables 1, 2). Sexual minority women scored lower on physical health compared to sexual minority men, *F* (1, 81) = 4.530, *p* < 0.001. Additionally, results showed that young older adults scored higher on the personal resiliency scale (i.e., RSCS), *F* (1, 81) = 7.205, *p* < 0.001, compared to old-old adults. Conversely, old-old adults scored higher on the experiences of discrimination and prejudice scale (i.e., DHEQ), *F* (1, 81) = 7.205, *p* < 0.001, compared to the younger group. Moreover, we found differences regarding coming out: Results showed that sexual minority men came out to all more frequently than sexual minority women, *χ*^2^(4) = 16.81, *p* < 0.01.

**Table 1:** Descriptive statistics and one-way analyses of variance: age differences.

Measures	Young older adults		Old-old adults		*F* (1, 81)	*p*
	M	DS	M	DS		
CO	3.81	1.27	3.61	1.09	0.37	0.54
DHEQ	0.53	0.56	1.44	1.33	18.75	0.00
RSCS	5.71	0.96	4.85	1.84	7.20	0.01
Physical Health	3.14	0.69	2.87	0.92	1.93	0.17
SES	4.11	0.75	3.89	0.85	1.14	0.29

CO, coming out; DHEQ, daily heterosexist experiences questionnaire; RSCS, resilience scale personal competence; SES, social economic status.

**Table 2:** Descriptive statistics and one-way analyses of variance: sexual minority categories.

Measures	Sexual minority women		Sexual minority men		*F* (1, 81)	*p*
	M	DS	M	DS		
CO	3.53	0.94	3.90	1.36	1.74	0.19
DHEQ	0.91	1.08	0.63	0.72	1.98	0.16
RSCS	5.57	1.44	5.50	1.14	0.06	0.81
Physical Health	2.86	0.89	3.21	0.62	4.53	0.04
SES	3.95	0.71	4.13	0.81	0.97	0.33

CO, coming out; DHEQ, daily heterosexist experiences questionnaire; RSCS, resilience scale personal competence; SES, social economic status.

### Bivariate correlation among key variables

To investigate relationships between variables, bivariate correlations across sexual minority categories were performed (Table 3). Regardless of gender, physical health was positively associated with personal resiliency (*r* = 0. 45) and coming out (*r* = 0.64) and negatively with experiences of discrimination (*r* = −0.68) and age (*r* = −0.38). Only for sexual minority women, findings highlighted negative significant correlations between age and physical health (*r* = −0.38), personal resiliency (*r* = −0.55), and coming out (*r* = −0.47), and a positive association with experiences of discrimination (*r* = 0.63).

**Table 3:** Pearson’s *r* between the variables considered in the study.

	1	2	3	4	5	6
1. Age	1	−0.15	−0.38*	0.63*	−0.55*	−0.47*
2. SES	−0.14	1	0.07	−0.20	0.32	0.02
3. Physical Health	0.20	0.20	1	−0.68*	0.59*	0.64*
4. DHEQ	0.11	−0.18	−0.37**	1	−0.81**	−0.59**
5. RSCS	0.03	0.13	0.45**	−0.49**	1	0.59
6. CO	0.27	−0.01	0.44**	−0.16	0.08	1

*p < 0.05 and **p < 0.01. Sexual minority women’s scores are above the diagonal, while sexual minority men’s scores are below the diagonal. SES, social economic status; DHEQ, daily heterosexist experiences questionnaire; RSCS, resilience scale personal competence; CO, coming out.

### Physical health of older LGB adults

The hierarchical multiple regression model was used to examine further how age, gender, socioeconomic status (SES), coming out, personal resiliency (i.e., RSCS), and experiences of discrimination and prejudice (i.e., DHEQ questionnaire) are related to physical health in older LGB adults (Table 4). Notably, before the analysis all the variables were standardized. A preliminary analysis indicated that the data met assumptions of linearity, normality of residuals, homoscedasticity, and multicollinearity. In the first step, age, gender, and SES were entered. Given that we aimed to explore the differences between experiences of discrimination, coming out, and personal competence, the three variables were used in the second step.

**Table 4:** Hierarchical regression analyses to predict physical health: coming out, personal resiliency, and experiences of discrimination and prejudice.

	B	SE B	*β*	*R* ^2^	Δ*R*^2^
Step 1				0.079	0.04
Sexual Minority Category	0.437	0.226	0.12		
Age	−0.09	0.11	−0.087		
SES	0.124	0.11	0.124		
Step 2				0.482***	0.441
Sexual Minority Category	0.301	0.178	0.146		
Age	0.075	0.092	0.075		
SES	0.045	0.086	0.045		
DHEQ	−0.289	0.120	−0.289*		
RSCS	0.252	0.111	0.252*		
CO	0.328	0.089	0.328***		

The tabled values for beta reflect *Bs* after step 2, *p < 0.05, **p < 0.01, and ***p < 0.001. High scores indicate: being sexual minority men, being over 70 years old, having higher SES, being out in the society, having a higher level of personal resiliency, having numerous experiences of discrimination and prejudice. Sexual Minority Category (0 = Sexual minority women, 1 = Sexual minority men); SES, social economic status; DHEQ, daily heterosexist experiences qestionnaire; RSCS, resilience scale personal competence; CO, coming out.

In the first step, the analyses revealed that SES, sexual minority category (sexual minority women VS sexual minority men), and age were not associated with physical health. Conversely, in the second step, findings showed that physical health was higher for older LGB adults who had had fewer experiences of discrimination and prejudice, were vastly out with their community regarding their sexual orientation, and reported a higher level of personal resiliency. Conversely, physical health was not associated with age and SES. The interaction effects between continuous variables and age, SES, and sexual minority category were not significant. The adjusted *R*^2^ for the whole model was 0.48.

## Discussion

The present study analyzed the relationship between physical health and experiences of discrimination and prejudice, coming out, and personal resiliency in older LGB Italian adults. Although the study population is smaller than in previous studies, the results seem to be in line with the literature, showing that sexual minority women reported lower levels of physical health compared to sexual minority men (Valanis et al., 2000; Fredriksen-Goldsen et al., 2013). Indeed, previous studies highlighted that sexual minority men, and specifically gay men, tended to pay more attention to their bodies and wellness to look younger and ageless (Slevin, 2010; Pistella et al., 2024).

Regarding the coming out process, results may show that sexual minority men tended to come out more frequently compared to sexual minority women. This result seems to differ from other studies that, conversely, found that sexual minority women tended to be more out compared to sexual minority men at older ages (D’Augelli and Grossman, 2001; Grov et al., 2006; Rosati et al., 2020). Given this difference, we can only speculate that this result could be interpreted considering the higher invisibility that sexual minority women live in the cis-hetero society and sometimes also in the LGBTQ+ communities as well (Traies and Munt, 2014; Munson and Cook, 2016; Rosati et al., 2021a). Moreover, considering the Italian context, the present result could be explained by the higher stigma and discrimination that older lesbian and bisexual women live in Italy because of the sexist and homo-lesbo-bi-transphobic culture (Baiocco et al., 2018b; Rosati et al., 2021a; Pistella et al., 2024).

Despite the modesty of the sample size, we found differences regarding age groups: younger old adults reported a higher level of personal resiliency and a lower level of discrimination than old-old adults. These findings align with the literature that explored past knowledge of discrimination in older cohorts (D’Augelli and Grossman, 2001; Rosati et al., 2021a). Indeed, past societies were more resistant in accepting and recognizing sexual minority groups, forcing them to hide their sexual identity and, consequently, promoting the interiorization of sexual stigma. Conversely, young older adults could have had quite more opportunities to live their lives and express their sexual identity, facing them to confront society and, consequently, finding strategies to affirm and protect themselves (Pistella et al., 2024).

Finally, results from the hierarchical multiple regression model showed that coming out, with higher levels of personal resiliency and fewer experiences of discrimination and prejudice, predicted higher physical health in older LGB adults, regardless of SES, age groups, and sexual minority categories. Again, these results aligned with the literature: Older LGB adults with fewer experiences of discrimination and stigma would have higher self-esteem, physical health, and well-being (D’Augelli and Grossman, 2001; Fredricksen-Goldsen and de Vries, 2019). In sum, the present findings showed that living in an affirmative context where being out without the fear of being discriminated is a protecting factor for aging well and being safe, particularly for older LGB adults.

## Limitations of the study

The present study could be considered part of a developing line of research investigating predictors of aging well in older LGB adults. Despite the relevance of the results, the study presents some limits. Firstly, the number of participants and the unbalance sample size within the two age groups limit the generalizability of the results. Accordingly, the present results could be interpreted as the first explorative evidence regarding the relationship between physical health, coming out, personal resiliency, and experiences of prejudice and discrimination among older Italian sexual minority adults. Future studies could further explore the relationship between the variables and improve the number of participants, specifically in the old-old adults’ group. Secondly, for the present study, we considered the personal competence dimension of the Resilience Scale (i.e., RCSC), focusing on self-reliance, independence, determination, invincibility, mastery, resourcefulness, and perseverance abilities. Considering the recognized role of resilience as a personality characteristic that moderates the effect of stress and promotes adaptation, future studies could also investigate the acceptance of self and life’s dimension of the RSCS scale to investigate further adaptability, balance, flexibility, and balanced perspective of life in older LGB adults.

Thirdly, a large part of studies on older LGB adults were conducted in WEIRD (Western Educated Industrial Rich and Democratic) countries, where, even with different nuances, LGB people are generally also recognized in terms of rights and developing visualization. Future research should also study the process of aging well in older LGB people in non-Western countries, where there are different social and material conditions and where there could be a different cultural cognition of aging (i.e., collectivistic cultures). Accordingly, based on a more intersectional perspective, it should be relevant to investigating aging well and considering other social and cultural oppressions, like ethnic background, ability, and gender identity. As an example, future research could explore the intersection of different aspects of one’s own identity (i.e., age, gender, sexual orientation, race, and ability) through the Identity Behavior Theory (Simons, 2021), which is concerned with the role that identity plays in the prediction of behavioral enaction, and that could be relevant in the specific topic of older LGB people aging well processes.

In line with other research (i.e., Rosati et al., 2021b), it could be relevant also to consider the experiences of older non-cisgender adults to offer a broader perspective on the process of aging well in sexual minority people. Moreover, future studies may consider the presence and strengths of social boundaries beyond the traditional conception of families and their roles in predicting physical and mental health in older LGB adults. The relevance of “families by choice” in LGBTQ+ communities is well understood in literature as a protective factor for adjustment and well-being (Uchino, 2009; Baiocco et al., 2023).

## Conclusion

In the last decades, in Western countries, it is well known that the global population is getting older significantly, and consequently, the number of older LGB adults is possibly increasing (Fredriksen-Goldsen and Muraco, 2010; Pistella et al., 2024). Thus, studying how to promote and guarantee good aging in older age is relevant in psychology. However, another essential component is considering how the aging process occurs for older LGB adults. Accordingly, the present study seems to aligns with other recent studies investigating predictors and obstacles of getting older in LGB communities, focusing on personal resources (e.g., personal resiliency) and social influences (e.g., experiences of discrimination and prejudice) that impact aging well.

The ways to create a safer and more affirming society could be different, starting from promoting inclusive policies through the visibility of older LGB adults in society and physical activity-related contexts. Again, another crucial point for reducing prejudice and discrimination against older LGB adults is working on the cultural competencies of both professionals and institutions (Baiocco et al., 2022; Antoniucci et al., 2023; Pezzella et al., 2023). Knowledge and awareness of LGBTQ+ issues are necessary for recognizing the unique needs and resources of LGB people in terms of age, gender, and sexual orientation for promoting a healthy aging process. Ongoing education and advocacy are essential components of this effort.

## Data availability statement

The raw data supporting the conclusions of this article will be made available by the authors, without undue reservation.

## Ethics statement

The studies involving humans were approved by Ethic Committee of the Department of Developmental and Social Psychology, Sapienza University of Rome, Italy. The studies were conducted in accordance with the local legislation and institutional requirements. The participants provided their written informed consent to participate in this study.

## Author contributions

RB: Conceptualization, Data curation, Formal analysis, Investigation, Methodology, Supervision, Writing – original draft, Writing – review & editing. CA: Investigation, Writing – original draft, Writing – review & editing. JP: Data curation, Formal analysis, Investigation, Methodology, Writing – original draft, Writing – review & editing. GA: Writing – original draft, Writing – review & editing. FA: Writing – original draft, Writing – review & editing. AB: Writing – original draft, Writing – review & editing. AC: Writing – original draft, Writing – review & editing. LF: Writing – original draft, Writing – review & editing. CF: Writing – original draft, Writing – review & editing. TP: Writing – original draft, Writing – review & editing. FR: Writing – original draft, Writing – review & editing. ST: Writing – original draft, Writing – review & editing. FL: Data curation, Formal analysis, Investigation, Methodology, Supervision, Writing – original draft, Writing – review & editing.
